# Potential of Boronic Acid Derivatization and Activity in Agrochemical Discovery

**DOI:** 10.3390/molecules30143018

**Published:** 2025-07-18

**Authors:** Liangshuo Ji, Jianxin Wu, Yachen Zuo, Wenqiang Gao, Jiyao Feng, Zhenhua Zhang

**Affiliations:** 1 College of Science, China Agricultural University, Beijing 100193, China; b20213100711@cau.edu.cn (L.J.); 2021310060411@cau.edu.cn (J.W.); s20243102373@cau.edu.cn (Y.Z.); 2 College of Plant Protection, China Agricultural University, Beijing 100193, China; gwenqiang@outlook.com (W.G.); jyfeng@cau.edu.cn (J.F.)

**Keywords:** boron-containing compounds, agrochemical, environmental stability, environmental safety

## Abstract

Since the approval of Bortezomib (Velcade^®^) by the U.S. Food and Drug Administration (FDA) in 2003, boron-containing drugs have successfully entered the global market, spanning therapeutic areas such as anticancer, antibacterial, and antifungal agents. Meanwhile, boron is an essential trace element for plant growth, and boronic acid has been widely used as plant resistance inducers and growth promoters. In 2024, the Fungicide Resistance Action Committee (FRAC) introduced benzoxaboroles as a new category of fungicides, which fully demonstrates the significant application potential of boron-containing compounds (BCCs) in the field of agricultural fungicides. Recently, studies on BCCs in agriculture have emerged continuously. Compared with the systematic reviews in the pharmaceutical field, those focusing on BCCs in agriculture remain absent. This review systematically collates BCCs with reported biological activities from the literature over the past 20 years, from the perspective of boron-containing building blocks. It mainly focuses on the potential of boronic acid derivatization and its activities in agrochemicals. Additionally, it covers the applications of boron-containing building blocks in pharmaceuticals, including their action mechanisms.

## 1. Introduction

Agrochemicals, including insecticides, fungicides, herbicides, and plant growth regulators, are crucial for global food production and safety. The classification system of their active ingredients is usually based on active building blocks as the core basis. In terms of insecticides, diamide insecticides have attracted much attention in recent years. Their action targets are ryanodine receptors and γ-aminobutyric acid receptors, and they have excellent control effects on lepidoptera pests [1]. Strobilurins, the most popular fungicides, are market-favored for being low-toxic, highly effective, broad-spectrum, and systemic. They have a wide range of control targets, almost covering all fungal diseases [2]. In the current global herbicide market, glyphosate, as a systemic herbicide, occupies the top position in market share with absolute advantage by virtue of its high efficiency, broad spectrum, and low-cost characteristics [3]. Regarding the composition of plant growth regulators, gibberellins have core functions such as promoting growth, breaking dormancy, and increasing fruit set rate, playing a key role in regulating the process of plant growth and development [4]. These active building blocks function by binding to specific proteins of target organisms (such as pests, pathogens, weeds) or crops, serving as the core framework for agrochemical research and development. The research and development of agrochemicals highly depend on innovative breakthroughs in active building blocks. Taking strobilurin as an example, fungicides with it as the core building block, such as azoxystrobin and pyraclostrobin, support nearly 20% of the global fungicide market. A single highly efficient building block can give rise to dozens of structural analogs [5,6]. However, due to the single mechanism of action and long-term large-scale use of existing mainstream building blocks, the problem of resistance has become increasingly prominent [7]. This challenge not only weakens the efficacy of existing building blocks but also forces researchers to seek novel core structures with more unique mechanisms of action.

It is worth noting that due to the special structures and mechanisms of action of BCC, they have shown great application potential in medical fields such as anticancer, anti-viral, and antifungal [8]. Additionally, it has long been discovered that boric acid has functions of promoting growth regulation, inducing resistance, and sterilization in the agricultural field, which has enabled its certain application in the early fungicidal field [9,10,11,12,13]. In 2024, FRAC officially classified benzoxaboroles as a new fungicide category (Group 43) [14]. This move indicates that the application potential of boron-containing active building blocks in the field of agricultural fungicides has been recognized by international authorities. The unique electronic structure and biological targeting of boron atoms provide new ideas for designing novel pesticides with multiple targets and high selectivity, and hold the promise of breaking through the resistance bottleneck of traditional building blocks [15]. This review systematically introduces the structures and action targets of BCCs in medicine from the perspective of boron-containing building blocks, and discusses the current development and challenges of boron-containing agrochemicals, aiming to provide theoretical references and strategic support for the creation of novel agrochemicals.

## 2. Boron-Containing Building Blocks and Targets

**Figure 1:**
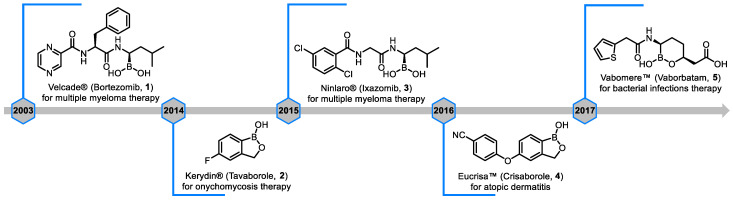
Timeline and structures of boron-containing drugs approved for marketing by the FDA [21].

In the long history of chemical synthesis and drug research, BCCs have gradually become a prominent focus due to their unique chemical properties and extensive application potential [16,17]. Initially, BCCs were first applied in the medical field, dating back to 1702 when BA solution was found to have a soothing effect. In 1956, Dr. Russell systematically reviewed the application of BA in treating skin diseases [18]. Since the 1990s, an increasing number of researchers have focused on the research and development of drugs using boron-containing building blocks [19,20,21,22,23]. In 2003, the first boron-containing drug Bortezomib (Velcade^®^) was approved for marketing by the FDA [24], marking the entry of boron-containing building blocks into a substantive stage of application in the pharmaceutical field. Subsequently, with the continuous deepening of research, five boron-containing drugs have successfully entered the market (Figure 1), covering anticancer drugs [25], antibacterial drugs [26], antifungal drugs [27], and treatment of atopic dermatitis [28].

**Figure 2:**
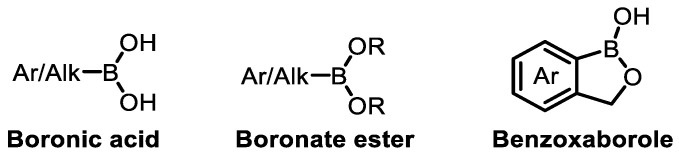
Structural classification of marketed or clinically studied boron-containing building blocks.

With the continuous development and the expansion of the application scope of BCC in the pharmaceutical field, and boron making increasingly significant contributions over the past 50 years, understanding their structural characteristics and action mechanisms becomes crucial. A growing number of BCCs are being considered as potential drugs. Among the currently marketed or clinically trialed boron-containing building blocks, based on their structures, they mainly include boronic acids, boronate esters, benzoxaboroles, etc (Figure 2) [29].

**Figure 3:**
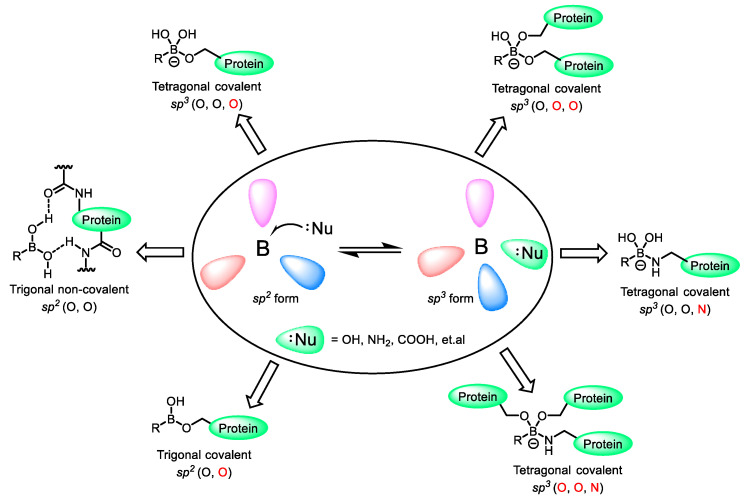
Boronic acid and its diverse binding modes with protein targets [21].

BCCs possess unique electronic properties. Due to the empty *p* orbital of boron, they exhibit certain electrophilicity. When accepting a pair of electrons from a nucleophile, they can easily transform from the *sp^2^* hybridization of a trigonal-planar structure to the *sp^3^* hybridization state of a tetrahedral structure [15]. Based on this property, organoboron compounds can form covalent adducts with nucleophiles such as amino acids (containing hydroxyl, amino, carboxyl groups) in target proteins, or sugars [30,31,32] (Figure 3). Moreover, the binding mode of BCC to enzymes is a special reversible covalent binding. It avoids both the off-target effects of non-covalent drugs and the high toxicity of covalent drugs. Additionally, the two hydroxyl groups of boronic acid provide four lone pairs and two hydrogen bond donors, thus offering six opportunities to form direct or water-mediated hydrogen bond contacts with key amino acid residues. Multiple hydrogen bonds can effectively enhance the affinity of BCC for proteins. Therefore, BCCs are of great significance for the development of new targeted drugs and the discovery of new target proteins [33]. The following lists compounds containing different boron-containing building blocks, as well as their targets and modes of action.

Most boronic acid compounds reversibly form covalent bonds with the hydroxyl groups of serine or threonine in target proteins, converting from a trigonal planar *sp^2^* hybridized structure to a tetrahedral *sp^3^* hybridized structure. Bortezomib (Velcade^®^) **1**, the first 20S proteasome inhibitor containing a boronic acid moiety, was approved by the FDA for the treatment of multiple myeloma. Initially, researchers screened proteasomes and discovered peptide aldehyde inhibitors. Co-crystal analysis with the target showed that they could covalently bind to nucleophilic threonine residues [34]. However, since aldehydes are not suitable for further drug development research, the pharmacophore was modified to a boronic acid analog and tested, and it was found to have high activity [35]. Ixazomib (Ninlaro^®^) **3**, as a second-generation 20S proteasome inhibitor [36], was approved by the FDA in 2015 [25] for the treatment of multiple myeloma. Its structure is similar to that of Bortezomib. The difference is that Bortezomib is administered as weekly injection [24], while Ixazomib is formulated as a citrate ester **9** to improve the metabolic stability and water solubility of the compound, making it an oral medication [25]. Therefore, it has better pharmacokinetic properties, is less likely to cause adverse side effects, and can also be used to treat certain patients whose tumors have developed resistance to Bortezomib [36].

**Figure 4:**
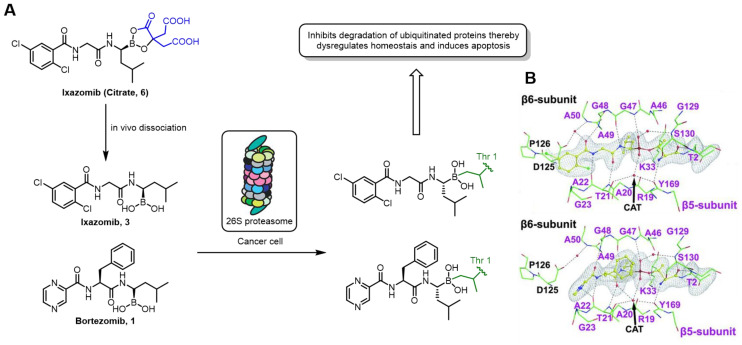
(**A**) Mechanism of action of Bortezomib and Ixazomib as 20S proteasome inhibitors. (**B**) Bortezomib and Ixazomib complexed into the active site of proteasome (pdb: 5LF1; pdb: 5LF3). Reprinted with permission from Ref. [38]. Copyright 2016, American Association for the Advancement of Science.

The 20S proteasome is the core molecular machine responsible for protein degradation within cells. It plays a crucial role in maintaining cellular homeostasis, regulating protein turnover, and participating in various physiological and pathological processes. Inhibiting the proteasome can reduce intracellular protein degradation, trigger endoplasmic reticulum stress, disrupt the cell cycle, and induce apoptosis [37]. Therefore, proteasome inhibition is considered an effective strategy for restricting cancer growth. Bortezomib and Ixazomib are both boronic acid inhibitors. Co-crystals show that both act on the 20S core particle of the 26S proteasome, inhibit the normal function of the proteasome by reversibly covalently binding to Thr1 of the β5 subunit (Figure 4B), and ultimately lead to the apoptosis of cancer cells [38].

**Figure 5:**
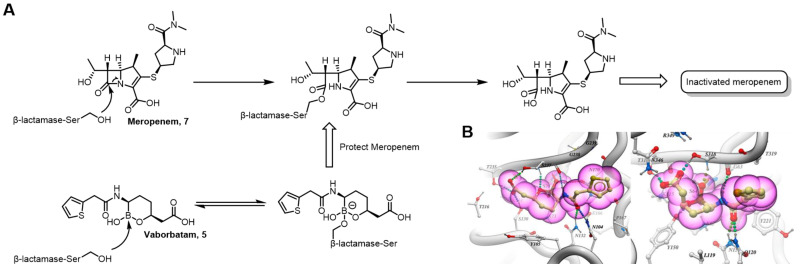
Mechanism of action of Vaborbactam as serine β-lactamase inhibitors. (**B**) Vaborbactam complexed into the active site of β-lactamase CTX-M-15 (pdb: 4XUZ) and β-lactamase AmpC (pdb: 4XUX). Reprinted with permission from Ref. [41]. Copyright 2015, American Chemical Society.

Serine β-lactamases in drug-resistant bacteria can hydrolyze β-lactam antibiotics, rendering them inactive. Vaborbactam (Vabomere™) **5** was approved for marketing by the FDA in 2017 and is used in combination with meropenem to treat bacterial infections [39,40]. Meropenem **14** is an inhibitor of bacterial cell-wall synthesis, while the boron atom of Vaborbactam binds to the serine residue (Ser) in the active center of β-lactamase through a competitive nucleophilic attack, forming a boronate ester intermediate. This process involves the formation of a reversible covalent bond between the empty *p* orbital of the boron atom and the lone pair of electrons on the oxygen of the serine hydroxyl group. This inhibits the hydrolysis of the β-lactam ring of meropenem by β-lactamase, thus protecting the antibiotic (Figure 5A). It is worth noting that Vaborbactam only targets class A and class C (Figure 5B) β-lactamases and does not bind to class B metallo-β-lactamases [41].

**Figure 6:**
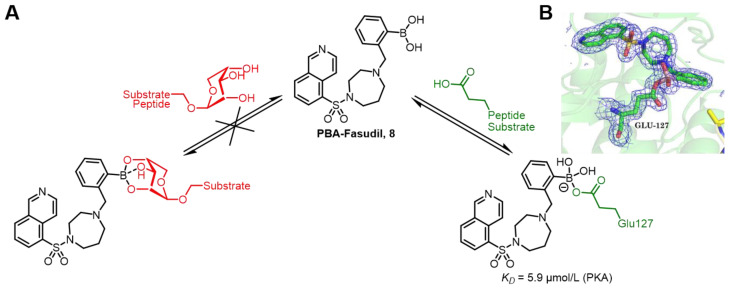
(**A**) PBA-Fasudil **8** inhibits PKA by a covalent bond to Glu127 in the active site. (**B**) PBA-Fasudil **8** attaches covalently to the carboxylate group of Glu127 and leads to a boronate complex. (pdb: 6ERT). Reprinted with permission from Ref. [42]. Copyright 2019, American Chemical Society.

In addition to reversible covalent binding with serine or threonine, the boronic functional group also interacts with glutamic acid containing a carboxyl group. A typical example is the discovery of protein kinase A (PKA) and its boron-containing inhibitor. PKA plays a crucial role in cell-cycle regulation and signal transduction, and dysregulation of its function may lead to the development of diseases. Fasudil is a known PKA inhibitor that exerts its inhibitory effect by binding to the furanose ribose of ATP in PKA. Klebe’s group drew inspiration from the spontaneous esterification of boronic acids with polyols and designed a boronic acid-modified PKA inhibitor, PBA-Fasudil **8**, aiming to bind to the cis-diols of furanose ribose. Unexpectedly, the crystal structure revealed that instead of the anticipated spontaneous esterification, it reacted with Glu127 in the binding pocket (Figure 6). This study provides a direction for the subsequent design of more effective bisubstrate-like inhibitors [42].

**Figure 7:**
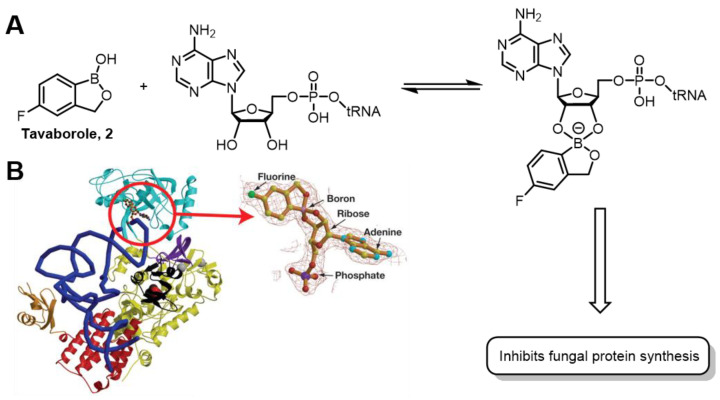
(**A**) Mechanism of action of Tavaborole **2** as LeuRS inhibitors. (**B**) Tavaborole **2** complexed into the active site of LeuRS (pdb: 2V0G). Reprinted with permission from Ref. [31]. Copyright 2007, American Association for the Advancement of Science.

Benzoxaboroles are cyclic boronic acid hemiesters called oxaboroles that are directly connected to a benzene ring [29]. Unlike boronic acids, the boron functional group of benzoxaborole is fused into a heteroaromatic ring. It exhibits better water solubility [19] and hydrolytic resistance [43] than boronic acids, and repeated clinical trials have demonstrated its low intrinsic toxicity [44]. These advantages make it an excellent boron-containing building block in drug design. Tavaborole (Kerydin^®^) **2**, the first FDA-approved drug containing a benzoxaborole scaffold, was launched in 2014 for treating onychomycosis (a fungal infection) [45]. Initially identified from a screen of boronic acid esters for antifungal activity [46,47], Tavaborole demonstrates broad-spectrum antifungal efficacy by inhibiting fungal leucyl-tRNA synthetase (LeuRS), which blocks protein synthesis and halts fungal growth [48]. LeuRS is an aminoacyl-tRNA synthetase. The enzyme catalyzes ligation of leucine to its cognate tRNA by sensing cytoplasmic leucine. Tavaborole exerts its antifungal action by inhibiting fungal protein synthesis. The compound inhibits protein synthesis by forming a covalent bond with LeuRS, thereby forming a tetrahedral covalent adduct (Figure 7A) with Ade 76 at the 3′ end of tRNA at the LeuRS editing site (Figure 7B), ultimately blocking the catalytic turnover of leucine [31].

**Figure 8:**
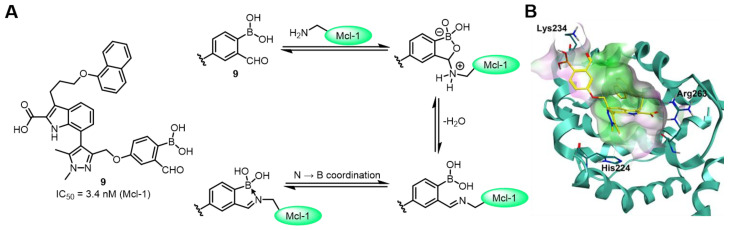
(**A**) Targeting of the noncatalytic ε-amino side chain of Lys234 in the Mcl-1 protein via iminoboronate with compound **9**. (**B**) Action stereogram of the interaction between compound **9** and Mcl-1. Reprinted with permission from Ref. [51]. Copyright 2016, Springer Nature.

The ε-amino group of lysine is protonated under physiological conditions and generally does not have the potential to form reversible covalent bonds with boronic acid functional groups [15]. Myeloid cell leukemia 1 (Mcl-1) is a key survival factor in a wide range of human cancers. As an anti-apoptotic protein, it maintains cell survival by inhibiting pro-apoptotic proteins and is highly expressed in multiple cancers. Developing drugs that can effectively inhibit Mcl-1 function is expected to break the anti-apoptotic barrier of tumor cells and reactivate the cell apoptosis program [49]. Inspired by reports of reversible covalent modification of lysine residues on protein surfaces by 2-carbonyl arylboronic acids [50], Grimster and Su’s group used molecular modeling to identify Lys234 of Mcl-1 as the optimal site for reversible covalent modification. They designed and synthesized a series of highly selective reversible inhibitors containing 2-carbonyl arylboronic acids. When modified with only a boronic acid or aldehyde group alone, the compounds showed only moderate inhibitory activity. However, when both boronic acid and aldehyde groups **9** were present, the inhibitory activity increased 112-fold (IC_50_ = 3.4 nM). Molecular docking studies revealed that the 2-carbonyl arylboronic acid undergoes reversible covalent modification with the side chain of Lys234 in Mcl-1 (Figure 8B). The authors also found that the boronic acid group activates the carbonyl group, making it more susceptible to nucleophilic attack by the ε-amino group of lysine to form an iminoboronate bond (Figure 8A). ^1^H NMR spectroscopy experiments confirmed that iminoboronate formation is favored at high concentrations, while the bond cleaves upon dilution, fully demonstrating the reversibility of this binding mode. These findings provide a new strategy for the development of Mcl-1 inhibitors [51].

**Figure 9:**
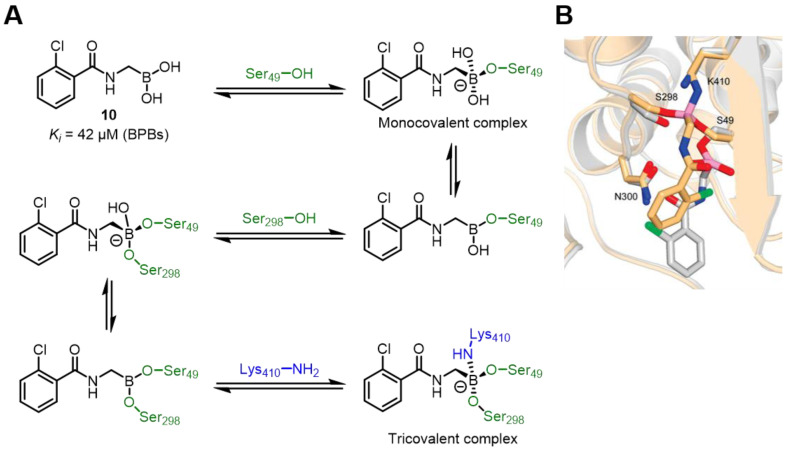
(**A**) Mechanism of action of compound **10** as PBPs inhibitors. (**B**) Overlap of monocovalent adduct (white) and tricovalent adduct (gold) of **10** with R39 (pdb: 2Y4A). Reprinted with permission from Ref. [53]. Copyright 2015, American Chemical Society.

Most boronic acids form monovalent complexes with enzymes, while the two hydroxyl groups of boronic acid also have the potential to interact with target proteins. Penicillin-binding proteins (PBPs) are a class of bacterial cell wall synthases that play a crucial role in maintaining the morphology and stability of bacterial cells [52]. Additionally, as a class of non-β-lactamases, in-depth research on PBPs can address the drug resistance problems caused by the overuse of β-lactamase antibiotics. Sauvage’s group studied boronic acid compounds with strong inhibitory effects on penicillin-binding protein R39, they found that the inhibitory effects of different boronic acids varied. Crystallographic results showed that boronic acid compound **10** formed monovalent and trivalent adducts at different active sites of the P39 protein. The monovalent adduct had boron only linked to the active serine; in the trivalent adduct, boron was linked to Ser49, Ser298, and Lys410 (Figure 9B). The authors emphasized that this trivalent binding mode is important for the inhibitory effect and can enhance the “residence time” of the inhibitor, providing ideas for the development of new antibacterial drugs [53].

**Scheme 1:**
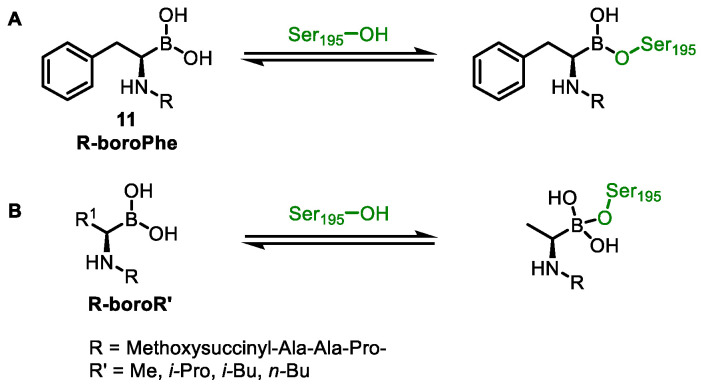
Schematic diagrams of the binding of different peptide boronic acid inhibitors to α-lytic protease complexes. (**A**) R-boroPhe. (**B**) R-boroVal, R-boroAla, R-boroNorleu, and R-boroIle.

When the pocket space of the target protein is relatively narrow, BCC often binds to the pocket in an unusual way. In a study on the complex structures of α-lytic protease and peptide boronic acid inhibitors [54], the compound R-boroPhe cannot fully enter the pocket due to the presence of the benzyl group, and forms a trigonal planar adduct with α-lytic protease (Scheme 1A). After replacing R-boroPhe **11** with amino acids with less steric hindrance such as R-boroVal, R-boroAla, R-boroNorleu, and R-boroIle, the compounds form tetrahedral adducts with α-lytic protease (Scheme 1B). Therefore, a thorough understanding of the structure of the substrate and its interaction mode with the inhibitor is the key to small-molecule design.

**Figure 10:**
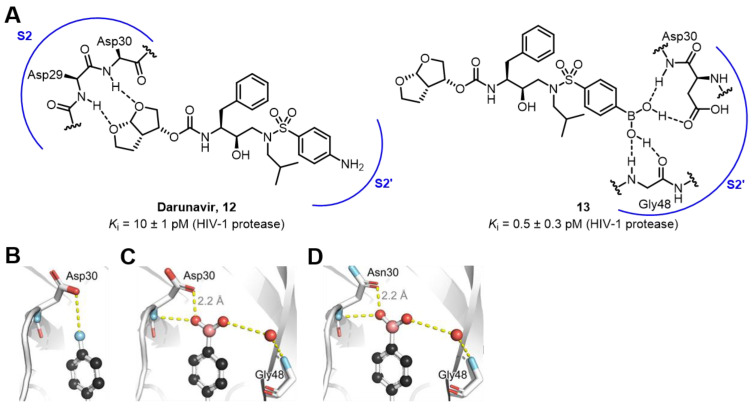
(**A**) Structures of darunavir **12** and compound **13** and interactions between **13** and the S2ʹ subsite of HIV-1 protease. (**B**) Interactions with darunavir **12** and the S2′ subsite of HIV-1 protease. (pdb: 4HLA). (**C**) Compounds **13** bound to wild-type HIV-1 protease. (pdb: 6C8X). (**D**) Compounds **13** bound to D30N HIV- 1 protease. (pdb: 6C8Y). Reprinted with permission from Ref. [59]. Copyright 2018, American Chemical Society.

As mentioned earlier, the two hydroxyl groups of boric acid provide four lone pairs and two hydrogen bond donors, thus offering the potential to form hydrogen bonds with key amino acid residues. HIV-1 protease is a crucial aspartic protease encoded by the HIV-1 virus gene and plays a vital role in the progression of AIDS [55]. Darunavir **12** is a highly active HIV-1 protease inhibitor [56,57]. Its bis-THF moiety can bind to the S2 subsite of the enzyme, achieving sub-picomolar affinity [58]. However, an ideal functional group for the S2′ subsite has remained elusive. None of the existing inhibitors can interact with both the main chain and side chain of Asp30 and the water molecule connected to the main chain of Gly48 simultaneously. Forest and Raines’s group exploited the ability of the two hydroxy groups of boronic acid to form multiple hydrogen bonds, which might be an ideal choice for the S2′ subsite. Therefore, they replaced the 4-sulfonylaniline moiety in darunavir with 4-sulfonylphenylboronic acid (Figure 10A). The inhibition constant (*K*i) of the resulting compound was 0.5 ± 0.3 pM, indicating a 20-fold greater affinity compared to darunavir. Moreover, its affinity for the drug-resistant D30N variant was comparable to that for the wild-type. X-ray crystallography analysis revealed that in the complexes formed by compound **13** with wild-type (Figure 10C) and D30N variant HIV-1 proteases (Figure 10D), the boronic acid participated in three hydrogen-bonding interactions. Among them, the BOH···O=C hydrogen bond exhibited the characteristics of a low-barrier hydrogen bond. This study highlights the value of boronic acid in the design of small-molecule ligands for drug-resistant proteins [59].

**Figure 11:**
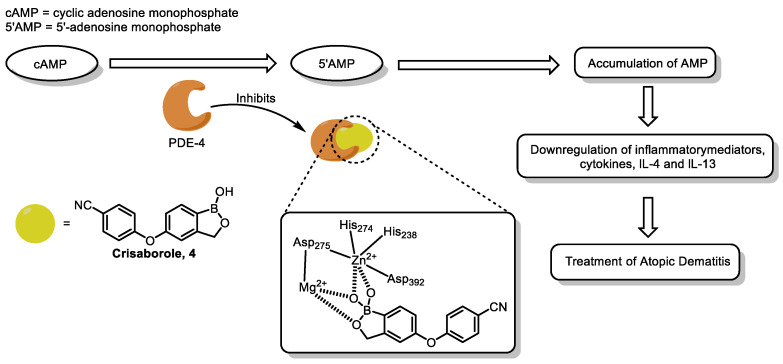
Mechanism of action of Crisaborole **4** as PDE-4 inhibitor. Reprinted with permission from Ref. [22]. Copyright 2022, Elsevier.

The binding mechanisms of BCC with target proteins are diverse, including not only the classical modes mentioned above, such as forming reversible covalent bonds or hydrogen bonds, but also new modes based on specific binding of special structures to metal centers [60]. Crisaborole **4** is a typical example [28], which binds to the bimetallic center of phosphodiesterase 4 (PDE-4) by competitively binding to the catalytic active site of PDE-4, inhibits the activity of PDE-4, and blocks the degradation of cyclic adenosine monophosphate (cAMP) by PDE-4. The increase in cAMP level further inhibits the nuclear factor-κB (NF-κB) pathway, reduces the release of pro-inflammatory mediators such as tumor necrosis factor-α (TNF-α), interleukin-12 (IL-12), and IL-23, exerts an anti-inflammatory effect, and alleviates the symptoms of diseases such as atopic dermatitis (Figure 11).

**Figure 12:**
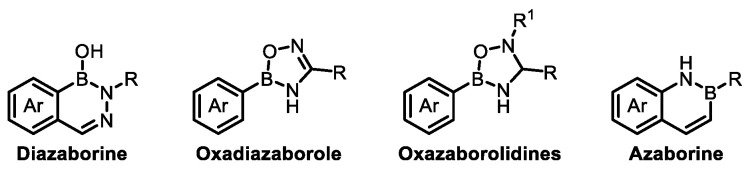
Structural classification of boron-containing heterocycles building blocks.

Regarding the research on BCCs, there has been a surge in development in recent years. In addition to those introduced above, other reported active boron-containing building blocks include diazaborine, oxadiazaborole, oxazaborolidines, azaborine, etc (Figure 12) [22]. These diverse boron-containing building blocks have demonstrated great application value and potential in the pharmaceutical field, bringing new possibilities for disease treatment. In fact, in the agricultural field, they also hold unlimited development prospects.

## 3. Boron-Containing Compounds in Agrochemical

From the existing reports on BCC in the medical field, both the exploration of boron-containing active building blocks and the discovery of related targets have witnessed in-depth development. These studies have great reference value for agrochemicals.

### 3.1. Boric Acid as Fungicides and Growth Regulators

**Figure 13:**
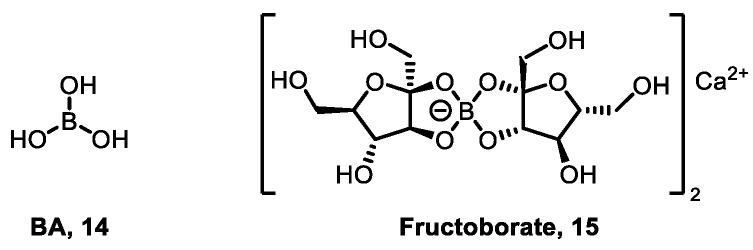
Examples of naturally occurring boron-containing compounds.

Boron is an essential element for vascular plants. It is involved in maintaining cell wall structure, cell membrane function, reproductive tissue development, nucleic acid synthesis, phenolic metabolism, and other processes [13]. For example, plants absorb BA **14** and form diester bond cross-links with rhamnogalacturonan II (RG-II) **15** [61] (Figure 13), directly maintaining cell wall integrity, which is an essential metabolic process for cell division and growth in vascular plants. Boron deficiency can also lead to abnormal development of root and shoot apical meristems, affect pollen tube growth and seed development, and influence the completion of the life cycle. It cannot be replaced by other elements.

Over the past 60 years, BA has been applied as a fertilizer to increase crop yields. For example, in a 2022 study, Hasan’s group found that the plant nutrient Alpha Boron Plus significantly reduced the incidence of potato common scab. The incidence of the disease decreased by 49.92% in terms of the number of infected tubers, 47.70% in terms of the weight of infected tubers, and 49.47% in terms of severity. Under field conditions, compared with other chemicals, Alpha Boron Plus increased potato tuber yield (18.07 tons/ha) by approximately 17.65%. This indicates that it has a dual effect of disease resistance and growth promotion [62].

**Figure 14:**
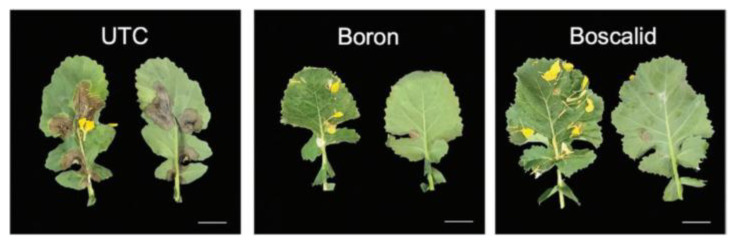
In vitro effects of BA or Boscalid on *S. sclerotiorum* in *B. napus* plant leaves. Reprinted with permission from Ref. [63]. Copyright 2024, Oxford University.

BA also has an inhibitory effect on common plant pathogens. For example, Belmonte’s group found that a 0.25% concentration of BA could significantly inhibit the mycelial growth of *S. sclerotiorum* in *B. napus*, and its inhibitory effect was close to that of the positive control fungicide Boscalid. When foliar spraying was carried out on *B. napus* leaves, it was found that the number of leaf lesions was reduced by 87% compared with the control (Figure 14). In subsequent mechanism studies, it was revealed that BA primed the plant’s defense response by inducing genes associated with systemic acquired resistance [63].

**Figure 15:**
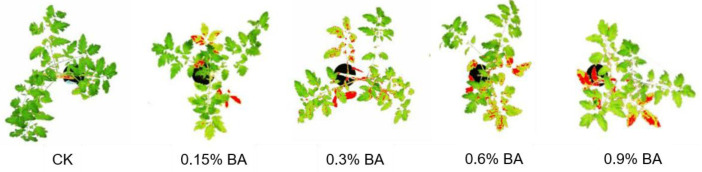
In vitro effects of BA on *A. alternata* in tomato plant leaves [64].

In a preventive experiment on tomato early blight caused by *A. alternata*, Martinko’s group tested the inhibitory effects of BA on *A. alternata* in vitro and in vivo, respectively. In the in vitro experiment, they found that a medium-concentration BA solution (0.06%) reduced mycelium formation by 45.2%, showing a certain fungistatic effect. In the in vivo experiment, different concentrations of BA (0.15%, 0.3%, 0.6%, 0.9%) were sprayed on the leaves of tomato plants. It was found that low concentrations (0.15%, 0.3%) reduced the lesion area by 83% and 68%, respectively, compared with the positive control, but the effect of high concentrations (0.6%, 0.9%) decreased, possibly due to phytotoxicity or pathogen adaptation (Figure 15). Therefore, BA can be used as an environmentally friendly fungicide and also as a boron for plants [64].

Extracting bioactive natural products from nature is an important means to explore new compounds. As a group of prokaryotes widely distributed throughout the world, cyanobacteria contain abundant antibacterial, antiviral, and other active components in their metabolites. The research team of Perveen extracted active components from *S. platensis*, *T. distorta*, and *M. aeruginosa* through different solvent systems, and found that when methanol/acetone/diethyl ether (5:2:1) was used as the extraction solvent, boronic acid, ethyl-, and dimethyl ester (with a content of 83.9%) in the extract of *M. aeruginosa* showed significant growth inhibitory effects on *F. solani* and *Cladosporium* sp. This finding provides potential candidates of natural active substances for the biological control of plant pathogenic microorganisms. This study not only expands the application prospect of cyanobacterial metabolites in agricultural disease control, but also provides an important scientific basis for the research and development of new natural product-oriented fungicides [65].

**Figure 16:**
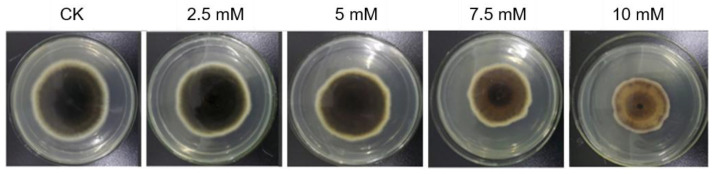
Effects of potassium tetraborate on the mycelial growth of *A. alternata* in vitro. Reprinted with permission from Ref. [66]. Copyright 2023, John Wiley and Sons.

Another study showed that boron in the form of potassium tetraborate could effectively inhibit mango black spot disease caused by *A. alternata*. Shi’s group found that potassium tetraborate could inhibit the mycelial growth of the mango black spot pathogen in a concentration-dependent manner, with an inhibition rate of 65% at 10 mM (Figure 16). Studies on its antifungal mechanism indicated that the potassium tetraborate treatments altered the expression of intracellular proteins involved in posttranslational modifications, protein turnover, and chaperones, as well as the mitochondrial proteins involved in the electron transport chain (ETC) [66].

In addition to simple boric acid and borates, Guo’s group pioneered the combination of iron and boron, two essential trace elements for plants. They found that high concentrations of iron (20 ppm) and boron (2 ppm) significantly reduced banana Fusarium wilt caused by *Fusarium oxysporum* f. sp. *cubense* (FOC) while promoting plant growth. Regarding the mechanism, the authors explained that high iron-boron concentrations inhibited FOC conidial germination and mycelial growth, reducing fusaric acid (FA) synthesis. Meanwhile, boron promoted mannitol accumulation in plants, which in turn decreased the phytotoxin production in infected plants, ultimately reducing the disease index attributed to phytotoxin virulence factors [67].

**Figure 17:**
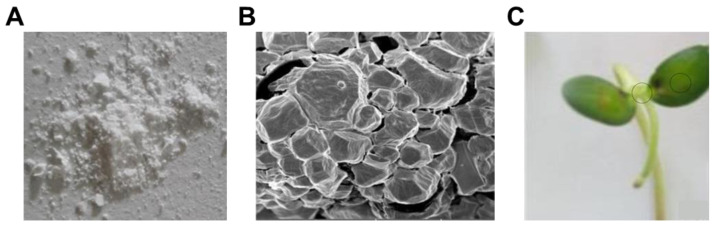
(**A**) Powder of SA/B complex. (**B**) SEM images of SA/B complexes. (**C**) Period of plants treated using the SA/B complexes [68].

Subsequently, Kabdrakhmanova’s group proposed an approach that combines the biological activity of natural organic acids, the antifungal properties of metal ions, and the structural optimization of coordination chemistry. Succinic acid (SA), a natural organic acid, is structurally similar to salicylic acid (a plant disease-resistance signaling molecule) and has the potential to induce systemic resistance. Drawing on previous studies regarding the inhibitory effects of complexes of SA with copper and silver against plant pathogens, this group innovatively incorporated boron into this coordination system (Figure 17A). Verified by FTIR and XRD, boron forms coordination bonds with the carboxyl oxygen atoms of SA to create a stable SA/B complex. A 0.1% solution of the synthesized SA/B complex exhibits significant antifungal effects against pathogenic fungi such as *F. oxysporum* and *A. alternata*, while promoting seed germination in the early stages of development. Moreover, after pre-sowing treatment of soybean seeds under laboratory conditions, the germination rate increased by 19.7% compared to the control samples (Figure 17C). These results suggest that the application of these complexes for pre-sowing treatment of agricultural crop seeds holds great promise [68].

**Figure 18:**
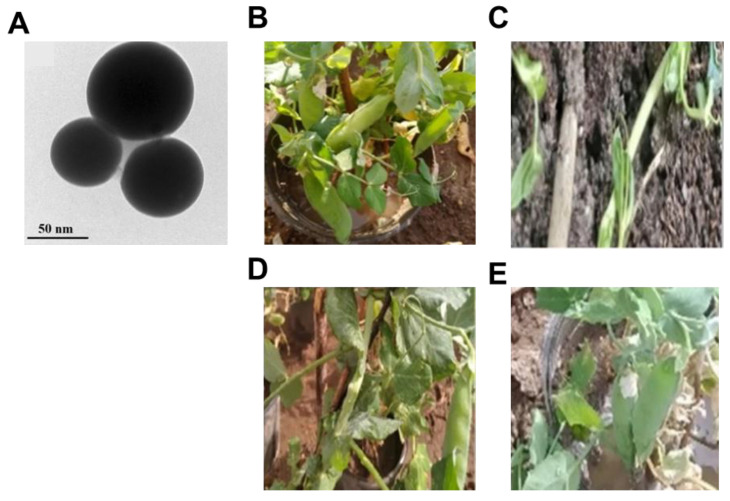
(**A**) HR-TEM images of the bimetallic ZnO-B_2_O_3_ nanoparticles. The effects of different treatments on pea plants infected with *P. irregulare*: (**B**) healthy control group; (**C**) infected control group; (**D**) infected group treated with bimetallic ZnO-B_2_O_3_ nanoparticles; (**E**) infected group treated with the chemical fungicide difenoconazole 25% [69].

Attia’s group prepared bimetallic ZnO-B_2_O_3_ nanoparticles to test their inhibitory activity against damping off disease of pea plants. The in vitro activity showed extremely strong antifungal activity (MIC = 0.01 μg/mL). Meanwhile, The in vivo experiment, ZnO-B_2_O_3_ nanoparticles significantly lessened the severity of the pea post-emergence damping off disease to 7.5% and provided a significant protection rate of 99.1%, which was superior to the control fungicide (difenoconazole, with a protection rate of 73.5%) (Figure 18B,E) [69].

### 3.2. Organic Boron Compounds

Although traditional boric acid has certain potential in aspects such as sterilization, promoting growth, and inducing resistance, long-term application of BA will lead to the gradually increasing problem of resistance. Moreover, the number of fungicide molecular targets that can be used for the creation of green pesticides is very limited. According to the statistics of FRAC, up to now, there are only more than 20 targets available for fungicide development. Most fungicides are developed for the same target. Among the fungicides widely used globally, 60% of the varieties only target three specific targets. In view of the fact that it is difficult to break through the bottlenecks of traditional boric acid in practical applications, many research groups have turned their attention to organoboron compounds.

**Figure 19:**
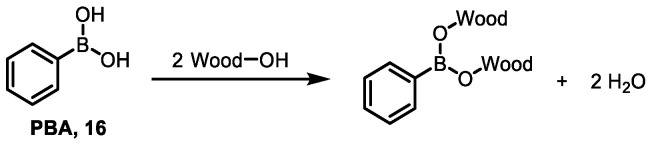
Possible structure of wood–PBA interaction.

Phenylboronic acid (PBA) **16**, as the simplest boron-containing organic compound, has been used as a fungicide for a long time. For example, Yalinkilic’s group impregnated wood with a low-concentration BPA solution (0.34%), which could increase the dimensional stability and biological durability of wood against *C. puteana* and *T. versicolor*. It also has the same termiticidal effect. The experiment also compared the effects of treating wood with BPA and BA. In the wood treated with PBA, most of the boron was fixed and not easily leached out. In contrast, the boron in the wood treated with BA could be completely leached out by water. This is probably because the connection formed between PBA and wood makes boron more stable to water leaching (Figure 19). The boron–oxygen bond (B-O) formed when BA binds to wood is relatively weak. The strength of the B-O bond in the BA-wood interaction is less than that of the bond formed between water molecules and wood through OH^−^ groups, so it is easily replaced by water [9].

**Figure 20:**
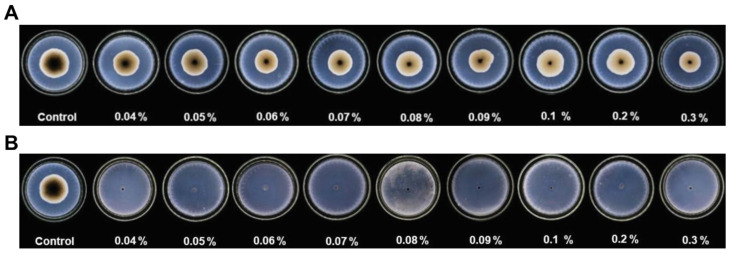
Inhibitory effects of different concentrations of (**A**) BA and (**B**) BPA (0.04–0.3%) on mycelial growth of *A. alternata*. [70].

As mentioned earlier, BA can prevent early blight of tomatoes caused by *A. alternata*, but its overall fungistatic effect is not satisfactory. When Đermić’s group replaced BA with BPA, they found that whether in in vitro mycelial inhibition experiments or in vivo prevention and control experiments of tomato early blight, the inhibitory activity of BPA against *A. alternata* was much higher than that of BA, and no plant diseases occurred at high concentrations (Figure 20) [70].

Subsequently, the research group turned their attention to plant pathogenic bacteria. In in vitro antibacterial experiments, PBA showed significant inhibitory activity against *P. syringae pv. tomato* and *E. amylovora*, and the bactericidal effect was concentration-dependent: it inhibited the growth of bacteria at ≤1.0 mg/mL and had a bactericidal effect at >1.0 mg/mL. Greenhouse experiments indicated that prophylactic application of PBA could reduce the symptoms of *P. syringae pv. tomato* infection in a dose-dependent manner (for example, the symptoms were reduced by 95% at a concentration of 2 times the MIC), and high concentrations of PBA (up to 1.5 mg/mL) had no significant toxicity to tomato plants. Due to its high efficiency, low toxicity, and environmental friendliness, PBA is expected to become a new type of plant antibacterial agent [71].

**Figure 21:**
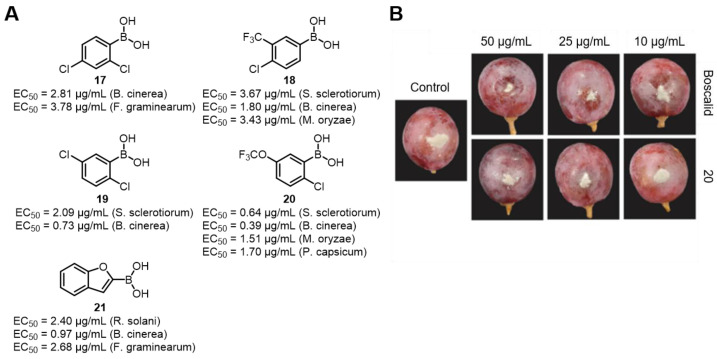
(**A**) Inhibitory activity of BPA derivatives against six phytopathogenic fungi. (**B**) In vivo activity against *B. cinerea* after compound **20** treatment. Reprinted with permission from Ref. [72]. Copyright 2023, John Wiley and Sons.

In addition to BPA, its derivatives also exhibit high-efficiency antifungal activities. Liu’s group screened 82 phenylboronic acid derivatives and found that 5 of them had high-efficiency antifungal activities against six plant pathogens (such as *B. cinerea*, *R. solani*, *M. oryzae*, etc.). They discovered that the introduction of electron-withdrawing groups such as Cl, CF_3_, OCF_3_, etc., onto the benzene ring could significantly enhance the activity, while electron-donating groups (such as CH_3_ and OCH_3_) had relatively low activities. Meanwhile, substituents like naphthalene, biphenyl, and benzofuran enhanced the activity, while heterocycles such as pyrimidine and pyridine had no obvious effects. Among the above five highly active compounds (Figure 21A), compound **20** (2-chloro-5-trifluoromethoxybenzeneboronic acid) has broad-spectrum antifungal activity. Its EC_50_ value against *B. cinerea* is 0.39 μg/mL, which is better than that of the positive control Boscalid (0.55 μg/mL) (Figure 21B). Subsequently, in the mechanism study, it was found that compound **20** inhibits fungal growth by disrupting cell membrane integrity, increasing membrane permeability, inducing the accumulation of reactive oxygen species (ROS), inhibiting the activities of antioxidant enzymes, and interfering with amino acid metabolism. Moreover, it has significant in vivo activity and low cytotoxicity to mammalian cells [72].

**Figure 22:**
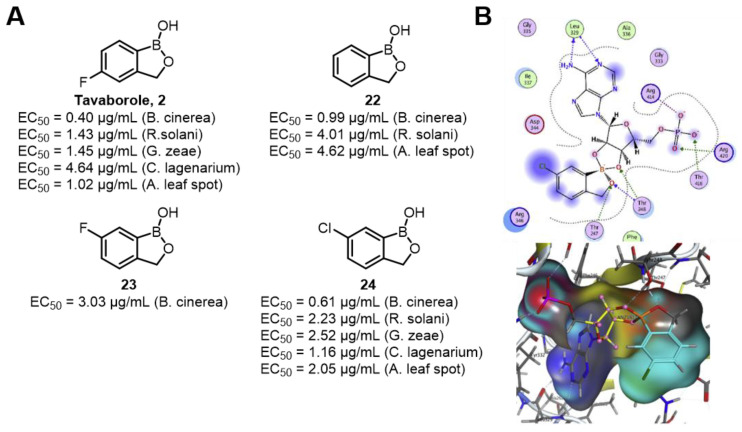
(**A**) Inhibitory activity of Tavaborole derivatives against six phytopathogenic fungi. (**B**,**C**) Planar graph and action stereogram of the interaction between compound **24** and *t*LeuRS [74].

In recent years, studies on active benzoxaboroles have emerged continuously. It should be noted that PBA typically binds to sugars in a reversible manner under physiological pH, relying on the formation of boronate esters with vicinal diols, though its binding affinity and selectivity are often limited, especially for non-reducing glycosides. In contrast, benzoxaboroles, with their unique heterocyclic structure, may exhibit different binding behaviors or even enhanced interactions with specific sugar moieties under similar conditions, though their primary mechanism of action in antifungal contexts is directed toward LeuRS rather than carbohydrate recognition [73]. The LeuRS targeted by benzoxaboroles is an essential enzyme in fungal protein synthesis, specifically involved in the aminoacylation of leucine-*t*RNA. Its function is highly conserved in pathogenic fungi and may become a new target that is not easy to induce drug resistance. Zhang’s group used Tavaborole as the lead structure and synthesized a series of derivatives containing the benzoxaborole structure. Through in vitro antifungal experiments, it was found that most of the compounds exhibited significant fungicidal activity against six plant pathogenic fungi at a concentration of 50 μg/mL (Figure 22). Among them, Tavaborle **2** showed particularly outstanding activity, and its antifungal effect was better than that of the positive control at some concentrations (for example, the EC_50_ values against *B. cinerea* were 0.40 μg/mL and 0.61 μg/mL, respectively). Molecular docking studies confirmed that compound **24** acts by targeting the CP1 domain of fungal LeuRS. The oxygen atom of the benzoxaborole ring forms hydrogen bonds with Thr247 and Thr248 of LeuRS, enhancing the binding stability [74].

**Figure 23:**
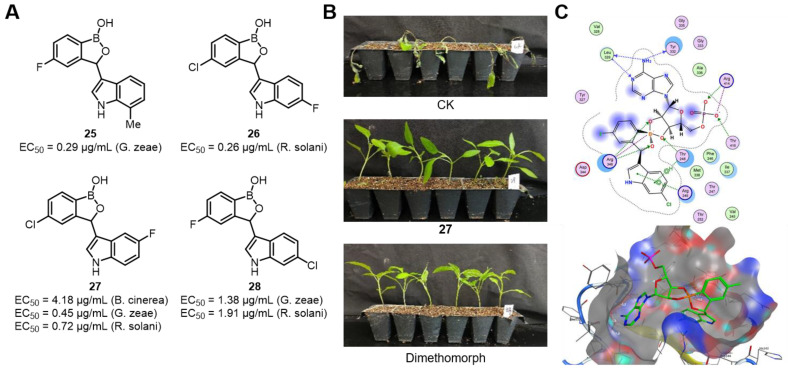
(**A**) Inhibitory activity of Benzoxaborole-containing Streptochlorin derivatives against phytopathogenic fungi. (**B**) In vivo activity against *P. capsici* after compound **27** treatment. (**C**) Planar graph and action stereogram of the interaction between compound **28** and *t*LeuRS. Reprinted with permission from Ref. [75]. Copyright 2023, American Chemical Society.

After discovering that benzoxaborole exhibits excellent inhibitory activity against phytopathogenic fungi, Zhang’s group drew inspiration from streptochlorin, a natural product with an indole skeleton. They introduced the benzoxaborole moiety into streptochlorin and designed and synthesized 46 streptochlorin derivatives containing benzoxaborole, aiming to discover novel derivatives with superior antifungal activity. In vitro antifungal activity tests indicated that most of the target compounds showed significant inhibitory effects against multiple phytopathogenic fungi (Figure 23A). Among them, compounds **25** and **26** demonstrated outstanding inhibitory effects against *G. zeae* and *R. solani*, with EC_50_ values of 0.29 μg/mL and 0.26 μg/mL, respectively, which were better than those of the positive controls. In vivo experiments showed that compound **27** had good activity against *G. zeae* and *R. solani*, with EC_50_ values of 0.45 μg/mL and 0.72 μg/mL. In the in vivo experiment, compound **27** showed a preventive efficacy of 95.5% against *P. capsici*, which was close to that of the control agent dimethomorph (100%) (Figure 23B).

Through 3D-QSAR studies, it was found that introducing appropriate electronegative groups at the 5-position of benzoxaborole and the 4,5-positions of the indole ring could improve the anti *B. cinerea* activity. After treating *G. zeae* with compound **27**, the mycelia collapsed and shriveled, indicating that **27** could damage the mycelial structure. It is speculated that it leads to the death of hyphae by destroying the cell membrane and cell wall. Through structural comparisons and literature reports, it is speculated that the target of streptochlorin derivatives may be LeuRS. Molecular docking showed that the oxygen atom on the benzoxaborole of compound **28** formed a hydrogen bond with Arg346, and Arg249 and Thr248 were connected to the aromatic ring of the compound through *π*–alkyl interactions (Figure 23C), explaining the necessity of the indole moiety [75].

**Figure 24:**
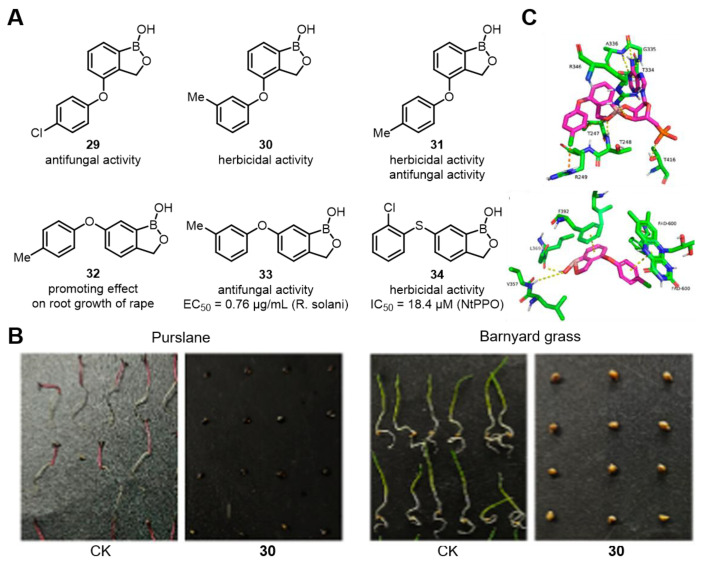
(**A**) Inhibitory activity of diphenyl-(thio)ether-containing benzoxaborole derivatives against phytopathogenic fungi and weeds. (**B**) Inhibitory effect of compound **30** on seed germination of purslane and barnyard grass. (**C**) Action stereogram of the interaction between compound **29** with *t*LeuRS and NtPPO. Reprinted with permission from Ref. [76]. Copyright 2025, American Chemical Society.

In addition to Tavaborole analogs, Zhang’s group also drew inspiration from the structure of crisaborole and designed and synthesized 30 novel diphenyl-(thio)ether-containing benzoxaborole derivatives (Figure 24A). In the in vitro antifungal activity tests, most of the target compounds showed excellent antifungal activity against six kinds of plant pathogenic fungi. For example, compounds **29** and **31** displayed 100% inhibition effects against three kinds of the tested plant pathogenic fungi under the concentrations of 50.0 μg/mL, and the EC_50_ value of compound **33** against *R. solani* was 0.76 μg/mL, significantly lower than that of Boscalid (1.20 μg/mL), which was used as the positive control.

Meanwhile, most of the compounds also demonstrated promising herbicidal activity. Compounds such as **30** and **31** showed effective control on purslane and barnyard grass. Moreover, compound **34** exhibited remarkable inhibitory activity against nicotiana tabacum protoporphyrinogen oxidase (NtPPO), with an IC_50_ value of 18.4 μM. Compound **32** also had a certain promoting effect on the root growth of rape. In addition, the authors evaluated the safety of compound **29**. The results showed that after treating cucumber plants with different concentrations of compound **29**, there were no significant effects on the fresh weight and plant height of the plants, demonstrating that compound **29** has the potential to become an environment-friendly pesticide.

LeuRS and NtPPO are important targets in the development of green pesticides. The authors docked the crystal structures of compound **29** with LeuRS and NtPPO to clarify the mode of action of the compound. For instance, Thr247 and Thr248 in LeuRS formed hydrogen bonds with the benzoborazole pentacyclic ring; in NtPPO, Val357 and Leu369 formed hydrogen bonds, and the two benzene rings had *π*−*π* stacking interactions with Phe392 and the large FAD molecule (Figure 24B), revealing the importance of the benzoborazole pentacyclic ring. This series of compounds has the potential to become dual-target fungicides and herbicides [76].

**Figure 25:**
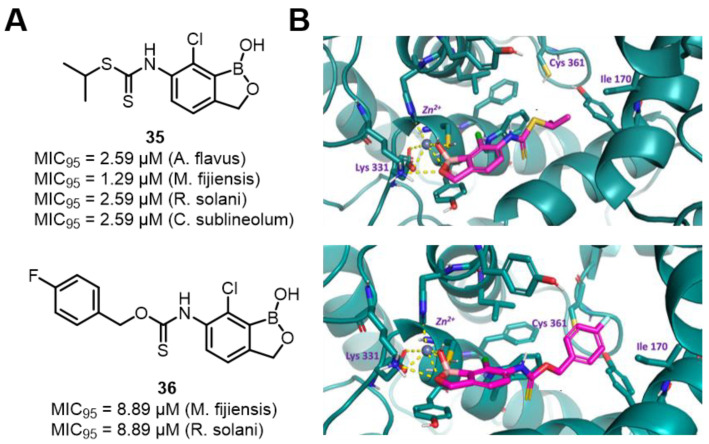
(**A**) Inhibitory activity of 6-thiocarbamate benzoxaboroles derivatives against phytopathogenic fungi. (**B**) Action stereogram of the interaction between compounds **35** and **36** with GGTase I. Reprinted with permission from Ref. [77]. Copyright 2020, American Chemical Society.

In addition to LeuRS, researchers are also attempting to identify new fungicidal targets for BCC. Protein prenylation plays a crucial role in eukaryotic cells. The geranylgeranylation modification catalyzed by geranylgeranyl transferase I (GGTase I) is essential for fungal signal transduction. Therefore, GGTase I can serve as a potential target for the development of novel agricultural fungicides. Cowen’s group designed and synthesized a series of 6-thiocarbamate benzoxaboroles and tested their antifungal activities against a variety of fungal pathogens. Compounds **35** and **36** were broadly active against multiple plant pathogens, with minimum inhibitory concentration (MIC_95_) values at or below 20 μM (Figure 25, A), which was comparable to the activities of conventional agricultural fungicides. However, the authors found that compound **35** had poor stability. It almost completely decomposed within 72 h at room temperature in phosphate buffer (Ph = 7.4), while compound **36** had stable chemical properties. Molecular docking indicated that compounds **35** and **36** bound to the active site of GGTase I (Figure 25B,C). The boron group interacted with the zinc atom and Lys331, inactivating the catalytic center of GGTase I. This article laid the foundation for further in-depth research on the antifungal mechanisms of the compounds and the development of novel agricultural fungicides [77].

**Figure 26:**
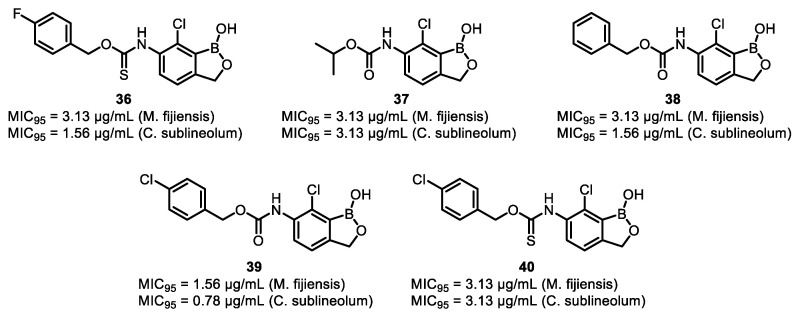
Evaluation of the antifungal activity of polysubstituted benzoxaboroles compounds.

Inspired by the above research, Pastoor’s group designed and synthesized a series of multi-substitution benzoxaboroles (Figure 26) and conducted a structure–activity relationship (SAR) study on them to identify potential fungicides that inhibit the fungal pathogens *M. fijiensis* and *C. sublineolum*. Through experiments, it was found that compounds **36-40** exhibited excellent antifungal activities, with MIC_95_ values in the range of 0.78-3.13 μg/mL against *M. fijiensis* and *C. sublineolum*. Mechanism of action studies showed that these compounds exerted their antifungal effects by inhibiting the activity of GGTase I, thereby affecting protein geranylgeranylation [78].

In recent years, many studies on BCC for agricultural use have focused on their fungistatic activity against plant pathogens and the underlying mechanisms. There are relatively few reports on their plant resistance induction and growth-promoting effects, and even fewer in the fields of insecticides and herbicides. However, the research gaps in these fields precisely provide vast space for cross-border innovation. Just as avermectin was transformed from an anti-parasitic drug [79] into a broad-spectrum insecticide [80,81] and thiabendazole was extended from a human antihelmintic [82] to a fungicide [83], BCC that have been successfully applied in the pharmaceutical field may serve as important sources of inspiration for the design of insecticidal/herbicidal active molecules with their unique mechanisms of action. However, it cannot be ignored that the current research and development of BCC for agricultural use face multiple challenges. There are objective realities such as the late start of research and the constraints on research and development investment due to the profit ceiling in the agricultural market, as well as technical challenges involving compound stability and environmental safety assessment. These issues urgently require cross-disciplinary teams to break the deadlock through collaborative innovation.

## 4. Current Challenges in Boron-Containing Agrochemicals

The prosperity of BCCs in the medical field stands in stark contrast to their slow progress in agriculture. The underlying reasons for this phenomenon can be summarized into three core challenges: First, there is a difference in research positioning and historical accumulation. For a long time, BCCs in agriculture have mainly been traditional boron fertilizers, with their functions centered on soil nutrient supplementation. The development of new active molecules started late, and a systematic research system has not been established yet. Second, industrial characteristics pose constraints. Unlike the high added value in the pharmaceutical field, the profit margin of agrochemicals is limited, making it difficult to cover the high costs of innovative research and reducing enterprises’ motivation to invest. Third, there is the problem of balancing multi-dimensional performance. Agricultural applications have more stringent requirements for compounds. It is necessary to optimize molecular design, formulation technology, and application methods to ensure both high efficiency and environmental safety (such as biodegradability and ecological toxicity), as well as field stability, which sets higher for research work.

### 4.1. Stability

**Scheme 2:**
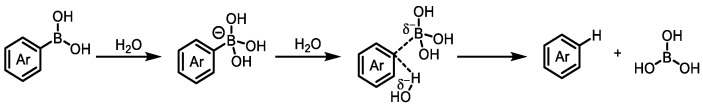
Hydrolysis mechanism of arylboronic acids.

First and foremost, the stability of boronic acid compounds requires attention. Compared with the acrylate-based agricultural compounds commonly used on the market currently, the stability of boronic acid is much lower, and it may degrade before binding to the target (Scheme 2) [84]. There are even studies proving that polyfluorinated phenylboronic acids show significantly decreased stability [85]. To address this issue, people prepare boronic acids into boronate esters to delay the hydrolysis process. However, common boronate esters such as boronic acid pinacol ester and boronic acid pinanediol ester are still prone to degradation in the natural environment. Benzoxaboroles can be treated as internal esters of the corresponding *o*-hydroxymethylphenylboronic acids. In comparison with other esters of boronic acids, the stability of the B-O bond is very high. It is completely resistant to hydrolysis: on the contrary, the corresponding *o*-hydroxymethylphenylboronic acid spontaneously dehydrates in water to form benzoxaboroles. Resistance to hydrolysis is also higher for B-C bond compared with boronic acid: benzoxaborole after refluxing for 3 h with 10% HCl gives, almost quantitatively, an unreacted compound, while BPA after 1.5 h decomposes in 90% [86]. Therefore, benzoxaboroles are more stable than BPA. Meanwhile, benzoxaborole compounds also have higher water solubility than BPA. As mentioned earlier, most of the boron-containing agricultural compounds developed in recent years have a benzoxaborole structure.

**Figure 27:**
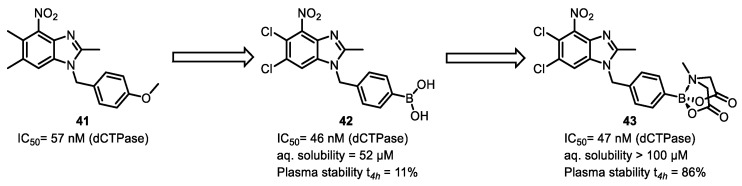
BMIDA can significantly improve the water solubility and stability of boronic acid compounds.

In addition, studies have shown that the tertiary amine group in *N*-methyliminodiacetic acid (MIDA) boronate coordinates with the empty p-orbital of the boron atom, preventing unnecessary reactions with external nucleophiles and reducing the hydrolysis rate of boronic acid compounds. This type of boronate can be stored stably at room temperature and does not undergo Suzuki coupling even after 28 h under anhydrous conditions [87]. Therefore, the MIDA group helps to improve the stability of boronic acid compounds. Meanwhile, research has indicated that MIDA-protected boronates can hydrolyze in biological media to produce the corresponding boronic acids, serving as a slow-release element [88]. For example, dCTPase pyrophosphatase 1 (dCTPase) is overexpressed in multiple carcinomas. Minguez’s group identified a lead compound **41** (IC_50_ = 57 nM) via high-throughput screening (HTS). Subsequent optimization led to the identification of a highly active boronic acid derivative **42** (IC_50_ = 46 nM). However, unfortunately, compound **42** only exhibited moderate aqueous solubility (52 μM) and low plasma stability (only 11% remaining after 4 h). To improve the absorption, distribution, metabolism, and elimination properties of compound **42**, they masked the boronic acid functionality with MIDA ester to obtain derivative **43**. It displayed greater aqueous solubility (> 100 μM), improved plasma stability (86% left after 4 h), enhanced cellular permeability, and comparable dCTPase inhibitory activity (IC_50_ = 47 nM) (Figure 27) [89].

To improve the stability of boronic acids, Burke’s group developed a new class of hyperstable tetramethyl *N*-methyliminodiacetic acid (TIDA) boronates in 2022. Through hyperconjugative and steric tuning, TIDA boronates exhibit better stability than the previously reported MIDA boronates. TIDA introduces hyperconjugation through tetramethyl substitution, enhancing the covalency of the N-B bond (redistribution of electron density) and suppressing hydrolysis. In the hydrolysis resistance experiment, TIDA boronate remained >99% after 6 h in THF/H_2_O-K_2_CO_3_ (60 °C), while MIDA boronate was completely hydrolyzed, indicating that this structure has strong hydrolysis resistance [90].

### 4.2. Synthesis Cost

**Scheme 3:**
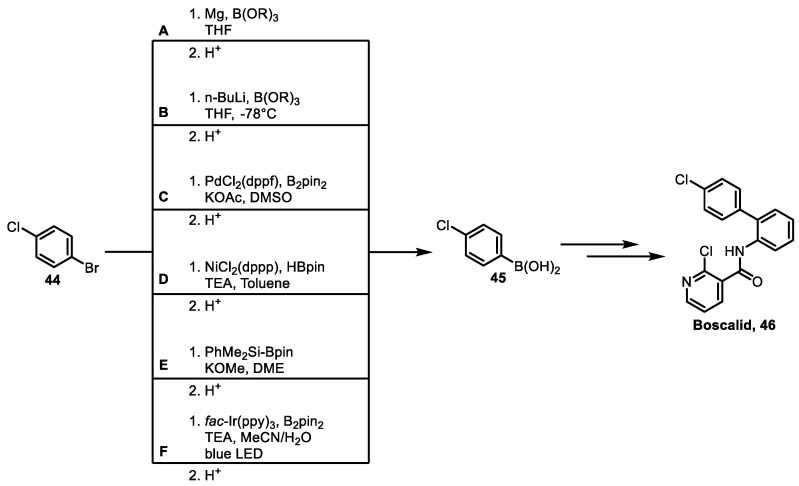
Key steps for synthesizing agricultural fungicide Boscalid: (**A**) Grignard reagent method; (**B**) organolithium reagent method; (**C**) palladium-catalyzed Miyaura borylation; (**D**) nickel-catalyzed borylation method; (**E**) metal-free catalyzed borylation method; (**F**) visible light-induced borylation method.

In addition to stability and environmental safety, the synthesis cost of agrochemicals is influenced by multiple factors due to their limited profit margins, such as raw material costs, synthetic processes, labor expenses, logistics, and transportation. For bulk agrochemicals, raw material costs typically account for 50–60%, while synthetic process costs only account for 25–30% [91]. Therefore, synthetic cost is also a critical factor in the synthesis of agrochemicals. As mentioned in the previous chapter, most currently active boron-containing agrochemicals are derivatives of phenylboronic acid or benzoxaborole. For phenylboronic acid derivatives, the key step in synthesis is the introduction of the boronic acid functional group. Taking the commercial pesticide Boscalid **46** as an example, the key intermediate of Boscalid is 4-chlorophenylboronic acid **45**, and its synthesis methods mainly include the following (Scheme 3): (A) Grignard reagent method; (B) organolithium reagent method; (C) palladium-catalyzed Miyaura borylation; (D) nickel-catalyzed borylation method; (E) metal-free catalyzed borylation method; and (F) visible light-induced borylation method.

The Grignard reagent method (Scheme 3A) usually involves preparing a Grignard reagent from 4-chlorobromobenzene **44** and magnesium turnings, followed by an in situ nucleophilic substitution reaction with alkyl borate at low temperature, and then quenching with hydrochloric acid to obtain 4-chlorophenylboronic acid **45** [92]. This method has a wide application range. The preparation of Grignard reagents is carried out at room temperature or under heating. The raw materials are cheap and easily available, and the operation is simple and safe, so it is usually used in large-scale production. Moreover, for relatively expensive bromoarenes, chloroarenes can be used as substitutes.

The organolithium reagent method (Scheme 3B) involves first performing a lithium-bromine exchange reaction between 4-chlorobromobenzene **44** and *n*-butyllithium to obtain substituted lithium benzene, then reacting with trialkyl borate, and hydrolyzing to obtain 4-chlorophenylboronic acid **45** [93]. The organolithium reagent method is widely used, with high yield and easy purification. However, organolithium is expensive and dangerous, and the reaction requires a low temperature of 78 °C, making it unsuitable for industrial production.

Miyaura borylation (Scheme 3C) involves the reaction of 4-chlorobromobenzene **44** with pinacol diboronate (B_2_pin_2_) under the catalysis of PdCl_2_ (dppf) to obtain the corresponding aryl boronate, which is then hydrolyzed to 4-chlorophenylboronic acid **45** [94]. This method is simple to operate and has a high yield, but the high cost of palladium catalysts makes it unsuitable for industrial production.

Since palladium catalysts are expensive, relatively inexpensive nickel catalysts have become good substitutes for palladium catalysts (Scheme 3D). Using triethylamine as the base and catalyzed by NiCl_2_ (dppp), 4-chlorobromobenzene **44** undergoes a coupling reaction with pinacolborane to obtain the corresponding boronate ester, which is then hydrolyzed to form 4-chlorophenylboronic acid **45** [95].

The metal-free catalyzed borylation method generally uses silaboranes as borylation reagents (Scheme 3E). In the presence of alkoxy bases, they react with 4-chlorobromobenzene **44** to form the corresponding aryl boronate esters, which are then hydrolyzed to 4-chlorophenylboronic acid **45** [96]. This method avoids the heavy metal residues caused by transition metal catalysis. However, the cost of borylation reagents limits the scale-up of this reaction.

The visible light-induced borylation method uses *fac*-Ir (ppy)_3_ as a photocatalyst (Scheme 3F). B_2_pin_2_ and 4-chlorobromobenzene **44** can obtain the corresponding aryl boronate esters in high yield under mild conditions, which are then hydrolyzed to 4-chlorophenylboronic acid **45** [97]. Meanwhile, owing to the development of continuous flow technology [98], the visible light-induced borylation method has great development potential in the future.

**Scheme 4:**
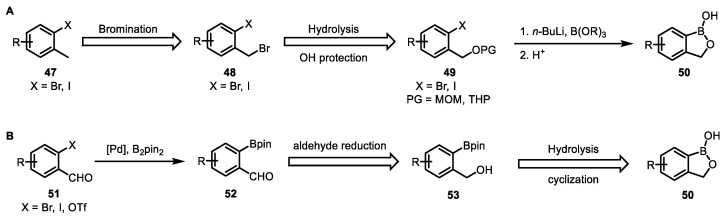
General synthetic routes for benzoxaboroles. (**A**) introducing a hydroxymethyl group into a boronic acid molecule. (**B**) introducing a boron group into benzyl alcohol.

Currently, the common methods for synthesizing benzoxaboroles can be roughly divided into two types [86]: (A) Firstly, by introducing a hydroxymethyl group into a boronic acid molecule (Scheme 4A) and bromination of the benzylic methyl of an *o-*bromo/iodo toluene derivative followed by substitution to generate benzyl alcohol. The hydroxyl group is generally protected via conversion to 3,4-dihydropyran or methoxy methyl ether. The boronic group is installed via Li-X exchange with butyllithium followed by addition of borate. Finally, the protecting groups are removed under acidic conditions resulting in intramolecular esterification. (B) Secondly, by introducing a boron group into benzyl alcohol (Scheme 4B), using *o*-formyl-substituted aryl halides or triflates, arylboronates are obtained after Miyaura borylation reduction in the formyl group produces an alcohol that coordinates boron. Hydrolysis of the pinacol ester under acidic conditions produces the final benzoxaboroles. Depending on the structure of the final compound, appropriate protection of the functional groups is carried out. Method A is of low cost. However, it requires the use of reactive reagents such as butyllithium, which increases the difficulty of the experiment. At the same time, precise control of the temperature and reaction time is needed, limiting its application in large-scale industrial production. Method B has mild conditions and high yields. Nevertheless, the use of a Pd catalyst leads to a relatively high synthesis cost, and there may be heavy metal residues.

**Figure 28:**
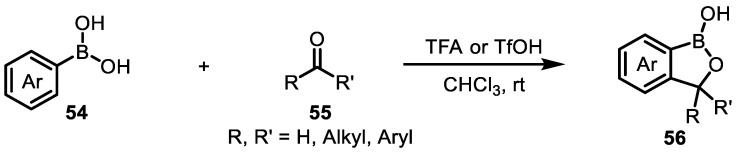
Synthesis of benzoxaborole compounds via *o*-oxyalkylation of arylboronic acids with aldehydes/ketones in the presence of brønsted acids.

For the method of introducing a hydroxymethyl group into a boronic acid molecule, a hydroxymethyl group is introduced into the boronic acid molecule. Li’s group developed a simple and efficient method (Figure 28) for the synthesis of benzoxaboroles **66** from arylboronic acids **54** and aldehydes/ketones **55** in the presence of a Brønsted acid. This methodology greatly simplifies the starting materials and reduces the number of reaction steps. The reaction can also be accomplished with acetals and ketals. The reaction has a very broad substrate scope and high practicability. Through the optimization of reaction conditions, trifluoroacetic acid (TFA) was determined to be the most efficient catalyst, and chlorohydrocarbon solvents were found to be suitable. Various arylboronic acids, aldehydes, ketones, acetals, and ketals can participate in the reaction. An intermolecular and an intramolecular Friedel−Crafts alkylation mechanism was proposed. Moreover, metals are not required for this transformation. This method may be very practical and beneficial in the synthesis of pharmaceuticals involving benzoxaboroles [99].

### 4.3. Environmental Safety

Given that organic boron-containing agricultural chemicals are still in the early stage of research, the lack of relevant statistical data leads to significant limitations in the environmental safety assessment of such chemicals. Among the currently known boron-containing agricultural chemicals with demonstrated biological activity, the toxicity studies on benzoxaborole derivatives are relatively the most in-depth. Taking Tavaborole as an example, human toxicity studies through multiple genotoxicity tests including the Ames assay, human lymphocyte chromosomal aberration assay, and rat micronucleus assay have shown that it has no potential for mutagenicity or clastogenicity; animal toxicity study data show that tavaborole did not exhibit genotoxicity or carcinogenicity in mice and rabbits [43].

However, since organic boron-containing agricultural chemicals are in the early stages of research and lack substantial statistical data, their environmental safety assessment remains limited. Studies have demonstrated that the primary toxicity of organic boron compounds originates from boric acid after degradation [100]. Extensive research on boric acid toxicity exists [101]; for example, a toxicological study on mice showed that high-dose boric acid (5000 ppm) caused teratogenicity and reproductive toxicity but did not induce genotoxicity or carcinogenicity [102]. Subsequent toxicity studies on rainbow trout (*O. mykiss*) revealed that low boron concentrations (<0.1 mg B/L) stimulated embryonic growth (with an 8% increase in body length), while high concentrations (>100 mg B/L) increased embryonic mortality (LC_50_ = 100 mg B/L). The U-shaped dose–response curve suggests that boron may be an essential trace element, with both deficiency and excess leading to adverse effects [103].

## 5. Conclusion and Outlooks

The vigorous development of boron chemistry has significantly expanded the application boundaries of BCCs in the agricultural field. This article systematically analyzes the multiple biological activities and mechanisms of action of boric acid and boron-containing building blocks. In the medical field, BCCs such as Bortezomib and Tavaborole demonstrate unique advantages in treating cancer, fungal infections, and inflammation by precisely targeting key sites like the proteasome and LeuRS, with their reversible covalent binding properties opening up new avenues for covalent-targeted drug development. In recent years, emerging phenylboronic acid derivatives and benzoxaboroles have demonstrated high-efficiency antifungal properties through mechanisms such as targeting key fungal metabolic enzymes and disrupting cell wall integrity, offering new approaches to address the challenge of resistance (Table 1). Although significant progress has been made in relevant activity studies, research on aspects like their environmental safety remains rarely reported.

Despite the promising prospects of boron-containing agrochemicals, their industrialization still faces three core challenges: stability, production cost, and environmental safety require further clarification and research. Agrochemicals impose more stringent multi-dimensional requirements on the cost–performance ratio, ecological toxicity thresholds, and field degradation characteristics of compounds. Future research should leverage target discovery techniques from the medical field. This can help establish a comprehensive research framework spanning from target identification to mechanism analysis. Simultaneously, breakthroughs in modular molecular design, biocatalytic synthesis processes, and nanoscale formulation technologies are essential to achieve synergistic optimization of stability, cost, and safety. With the deep integration of boron chemistry, chemical biology, and plant protection, the development of a cluster of novel boron-containing agrochemicals is anticipated. These agrochemicals are characterized by high efficiency, low toxicity, environmental friendliness, and innovative action mechanisms, and will provide disruptive solutions for constructing global agricultural green prevention and control systems and promoting sustainable development.

## Figures and Tables

**Table 1:** Boron-containing drugs in clinical trials.

Field	Compound Type	Protein Target	Mechanism and Solved Problems
Medicine	Peptide Boronic Acid	Proteasome; β-lactamase; PBPs	Treats multiple myeloma; Treats bacterial infections; Resolves bacterial drug resistance
	Benzoxaborole	LeuRS; PDE-4	Treats onychomycosis; Treats atopic dermatitis
	Phenylboronic Acid Derivatives	PKA; Mcl-1; HIV-1	Uncovers the covalent bonding mechanism of boronic acid and Glu; Addresses non-covalent Mcl-1 inhibitor off-target effects; Improves Darunavir’s HIV-1 protease targeting efficiency
Agriculture	Boric Acid	NPR3	Induces plant resistance
	Borates	POD; PPO	Enhances plant disease resistance
	Phenylboronic Acid Derivatives	POD; PPO	Disrupts cell membrane integrity Affects redox balance Interferes with amino acid metabolism
	Benzoxaborole	LeuRS; NtPPO; GGTase I	Develops novel antifungal agents and addresses fungicide resistance issues

## Data Availability

Not applicable.
